# GazeHRNet: Head-Centric Spatial Encoding and Gaze-Aware Feature Interaction for Gaze Target Detection

**DOI:** 10.3390/s26154799

**Published:** 2026-07-28

**Authors:** Tianxiang Nan, Chenglizhao Chen, Xi Chen, Zhi Li, Xiangyu Wei, Xinyu Liu

**Affiliations:** College of Computer Science and Technology, China University of Petroleum (East China), Qingdao 266580, China; 2307030119@s.upc.edu.cn (T.N.); cclz123@163.com (C.C.); 2307030115@s.upc.edu.cn (X.C.); 2307030120@s.upc.edu.cn (Z.L.); 2307030117@s.upc.edu.cn (X.W.)

**Keywords:** gaze target detection, head-centric encoding, gaze-aware attention, contrastive representation learning, vision foundation model

## Abstract

Gaze target detection requires understanding where a person is looking by jointly reasoning about the gazer and the surrounding scene. While recent methods have benefited from powerful pretrained visual backbones, they often treat gaze prediction as a generic localization problem and overlook a key property of the task: the target should be interpreted in relation to the person’s head. This limits their ability to model direction, distance, and head-scene dependencies in a unified manner. We propose GazeHRNet, a head-centric reasoning framework for RGB-based gaze target detection. Instead of relying on absolute image coordinates or auxiliary geometric inputs, GazeHRNet represents the scene from the gazer’s perspective through Head-Centric Polar Encoding and organizes visual features by their spatial relevance to the head via Head-Aware Attention Routing. It further combines coarse spatial reasoning with fine-grained anisotropic heatmap prediction, enabling reliable target localization under cluttered scenes and varying head positions. Experiments on GazeFollow and VideoAttentionTarget show that GazeHRNet achieves 0.952 and 0.929 AUC with L2 distances of 0.102 and 0.103, respectively, using only RGB input and 3 M trainable parameters. Cross-dataset evaluation further demonstrates improved robustness and generalization across different scenes and subject distributions.

## 1. Introduction

Understanding where people are looking is central to human social cognition. From early infancy, humans follow others’ gaze to infer intentions and establish joint attention [1]. Gaze target detection aims to predict the image location at which a person is looking, given their head position. It serves key applications including human–robot interaction [2,3], autonomous driving [4], and social behavior analysis [5,6]. It also supports augmented reality [7]. Unlike saliency detection, this task must reason about a specific person’s viewing direction conditioned on their head. It requires jointly modeling head pose, scene layout, and the geometric relationship between them.

This task has evolved through three stages. Early methods use dual-pathway architectures that encode the scene and a cropped head patch separately [8,9,10,11]. Subsequent works enrich this paradigm with depth maps [12,13], 3D gaze cones [14], object detections [15], and Transformer decoders [16,17]. Some methods exploit interaction patterns in pretrained ViT attention maps [18]. More recently, frozen pretrained backbones serve as unified feature extractors with lightweight task heads [19]. This paradigm achieves strong performance with far fewer trainable parameters. Yet across all three stages, the task-specific adaptation of these models often requires careful architectural design to fully exploit their rich representations.

Gaze prediction is inherently spatial reasoning centered at the head. A patch’s relevance depends on its direction and distance relative to the head, not its absolute coordinates. A patch at the same relative position carries identical gaze geometry regardless of where the head is. Yet all existing methods use task-agnostic positional encodings that represent only absolute locations. None encodes head-centric direction or distance. The feature interaction stage has a similar gap. Head tokens carry gaze direction cues. Scene tokens contain potential targets. Background tokens mostly contribute noise. Recent work has shown that pretrained ViT attention implicitly contains head-scene interaction patterns [18]. But these implicit patterns are not designed for gaze. They do not know which tokens belong to the head and which are background. No existing method explicitly biases attention weights based on head spatial membership. Backbone features also contain gaze-irrelevant information that should be filtered beforehand.

The supervision and representation sides have parallel gaps. Most methods use a single isotropic Gaussian heatmap as the training target. This assumes symmetric spatial uncertainty around the gaze point. It also provides no global spatial guidance for coarse localization. Gaze target annotations often exhibit directional variance [20], which isotropic labels cannot capture. Patch-level distribution prediction [20] and dual regression [21] have been explored separately. But no prior work combines coarse distribution with anisotropic fine-grained supervision. Beyond output-level supervision, the intermediate representations also lack structural constraints. Samples with similar gaze behaviors may be far apart in feature space. This limits generalization beyond the training distribution.

We propose GazeHRNet to address these gaps. It is a head-centric framework built on frozen pretrained backbones. Every component follows one principle: gaze prediction is spatial reasoning centered at the head. The main contributions are as follows:

- We propose Head-Centric Polar Encoding (HCPE). It encodes each token’s direction and distance relative to the head center through multi-frequency sinusoidal functions. This injects a geometric prior aligned with the gaze projection process. HCPE complements binary head prompting. Head prompting marks which patches are the head. HCPE describes how every patch relates to the head spatially.
- We design a two-stage gaze-aware feature interaction mechanism. The Gaze-guided Relation Sparse Propagation (GRSP) uses content-dependent similarity gating to suppress task-irrelevant redundancy before the Transformer. Unlike fixed linear adapters, GRSP selects token connections based on actual feature content. The Head-Aware Attention Routing (HAAR) explicitly restructures self-attention with a global [GAZE] token, head-guided bias, and local background suppression.
- We construct a coarse-to-fine spatial supervision framework. The Patch Distribution Predictor (PDP) predicts a gaze distribution over a coarse 8×8 grid with an outside token. The outside token unifies in-frame and out-of-frame prediction within a single distribution. Anisotropic Heatmap Supervision (AHS) uses an elliptical Gaussian ($\sigma_{x}\neq \sigma_{y}$) for fine-grained labels.
- We propose Head-Relative Rank-N-Contrast Learning (HR-RNC) for the [GAZE] token. It uses head-relative gaze vectors as the label space to remove absolute position bias. It employs a ranking-based contrastive loss [22] to preserve ordinal structure without hard thresholds. HR-RNC is a training-only loss with zero inference overhead.

We evaluate GazeHRNet on GazeFollow [10] and VideoAttentionTarget [11]. With only 3 M trainable parameters (alongside a frozen DINOv2 backbone) and RGB-only input, GazeHRNet achieves 0.952 and 0.929 AUC on GazeFollow and VideoAttentionTarget respectively, outperforming the compared computational models including those relying on depth, pose, or object detection. The source code and pre-trained models are publicly available at https://github.com/Zhikanyoumai/gazehrnet (accessed on 16 July 2026). Cross-dataset evaluation on three unseen benchmarks further suggests strong generalization. Ablation studies verify that each proposed module contributes meaningfully to the overall performance. Furthermore, by advancing the software-level perception capabilities of ubiquitous RGB cameras without requiring dedicated multi-sensor hardware, this lightweight and robust framework aligns well with the scope of intelligent sensing systems.

## 2. Related Work

### 2.1. Gaze Target Detection

Gaze target detection predicts the 2D location at which a person is looking in a scene image. The task was first formalized with a dual-pathway architecture that processes scene and head features separately [8]. Head position encoding as a spatial map fused with scene features through attention improved this paradigm [9]. The framework was extended to video with temporal modeling and the VideoAttentionTarget benchmark [11].

Auxiliary information then enriched this framework. Dual attention combining field-of-view and depth cues was proposed for 3D geometric reasoning [12]. Sight-line-guided gaze cones in 3D space improved target localization [14]. A modular architecture integrating RGB, depth, and pose showed that geometry alone yields competitive results [13]. ESCNet [23] incorporated 3D scene understanding. These cues provide useful priors but add complexity and error propagation.

Transformer architectures brought new approaches to gaze target detection. An end-to-end decoder was proposed to jointly detect heads and predict targets [16]. Object-level reasoning was integrated through head-object association [15]. Sharingan [24] enabled multi-person prediction in one forward pass. GazeHTA [17] used a pretrained diffusion model as a feature extractor. Dual regression combining coordinate and heatmap supervision reduced model complexity [21].

Frozen pretrained backbones recently emerged as unified feature extractors [19]. This paradigm uses lightweight heads on general-purpose features with far fewer parameters. GazeSeg [25] extended this direction to pixel-level gaze segmentation. Multi-view GTE [26] extended the task to multi-camera settings with cross-view geometric reasoning through epipolar constraints. Despite steady progress, open gaps remain in spatial encoding, attention design, supervision, and representation learning for single-view gaze target detection in the wild.

### 2.2. Gaze-Driven Human–Computer Interaction

Beyond benchmark-oriented gaze target detection, gaze information has been increasingly integrated into practical human–computer interaction systems, including assistive communication, webcam-based interaction, attention-aware interfaces, and AR/VR object selection [27,28]. Recent reviews indicate that the deployment of gaze-based interfaces depends not only on spatial estimation accuracy but also on calibration requirements, robustness to environmental variations, response latency, privacy, and user experience [29]. Accordingly, application-oriented studies commonly evaluate task completion time, selection error, dwell behavior, interaction efficiency, and subjective usability rather than gaze-localization metrics such as AUC and L2 distance [30]. These systems, therefore, address a different level of the gaze-processing pipeline from the present work. GazeHRNet focuses on the upstream perception problem of localizing a person’s gaze target from an RGB scene and a head bounding box. Its scene-level gaze prediction could provide a visual attention signal for attention-aware interfaces, human–robot collaboration, intelligent manufacturing [31], assistive control, and object selection in immersive environments. Nevertheless, integrating the proposed framework into complete interactive systems and evaluating it through real-time user studies and application-specific metrics remain important directions for future research.

### 2.3. Spatial Representation in Gaze Prediction

How spatial information is encoded strongly affects gaze prediction accuracy: Most methods rely on absolute positional encodings such as learnable embeddings [32] or 2D sinusoidal patterns. These represent a patch’s location in the image but carry no information about its relationship to the gazer. Relative position biases encode pairwise spatial offsets between tokens [33], but remain task-agnostic. GA3CE [34] explored a direction-distance decomposition encoding for unconstrained 3D gaze estimation, transforming scene context into a body-centric coordinate system. However, its encoding targets 3D body keypoints and object positions rather than 2D patch-level features anchored at a head bounding box. No existing method in gaze target detection encodes head-centric direction and distance for every scene token.

Beyond positional encoding, spatial reasoning also occurs in the attention mechanism. ViTGaze [18] showed that pretrained ViT self-attention implicitly contains head-scene interaction patterns useful for gaze following. This insight is valuable, but these implicit patterns arise from general pretraining, not gaze-specific design. They do not distinguish head regions from background. Task-general attention modifications such as deformable attention and mask-conditioned attention constrain spatial scope but do not inject head-based spatial structure into the computation. No existing method explicitly biases attention weights based on head spatial membership.

Feature adaptation is another relevant dimension. General-purpose backbone features contain broad visual information, and standard approaches such as low-rank matrices and learnable prompt tokens apply fixed transformations regardless of input content. However, content-dependent filtering based on inter-token similarity has not been explored for scene-level gaze target detection, where the filtering must operate on full-scene patch tokens before person-specific conditioning.

Self-supervised models like DINO [35], DINOv2 [36], MAE [37], and SAM [38] provide strong general features. With lightweight heads, they achieve competitive results on depth estimation and segmentation [36,39]. Adapting these features effectively for gaze-specific spatial reasoning remains an open problem.

### 2.4. Supervision and Representation Learning

The standard supervision for gaze target detection is a pixel-level heatmap. An isotropic Gaussian centered at the gaze point serves as the training label [8,11]. This design assumes circular spatial uncertainty around the target. Annotation studies suggest that gaze target variance is often directional [20]. Isotropic labels cannot capture this asymmetry.

Some works have explored richer supervision signals. Patch-level gaze distribution prediction provides coarse spatial guidance [20]. Dual regression combines coordinate prediction with heatmap regression [21]. ADGaze [40] observed that gaze features exhibit anisotropic structure across pitch and yaw directions and proposed elliptical label distributions for fine-grained gaze direction regression. Multi-scale supervision is widely used in dense prediction, but in gaze target detection, coarse-grained and fine-grained supervision have not been combined with anisotropic labels in a unified framework. Applying anisotropic spatial priors to heatmap-based GTE supervision remains unexplored.

Representation learning offers another angle. Contrastive learning has succeeded in self-supervised and supervised settings [41]. But standard contrastive losses use binary positive/negative pairs and cannot capture graded similarity in regression targets. The Rank-N-Contrast framework preserves ordinal structure without hard thresholds [22]. In gaze research, DCGaze [42] employs differential contrastive training with vision-language models, using pairwise relative gaze relationships rather than per-sample labels. CGaG [43] applies collaborative contrastive learning to disentangle gaze-relevant features from domain-specific factors. However, these methods target gaze direction estimation. Applying contrastive representation learning to gaze target detection, with labels that account for head position, remains unexplored.

## 3. Method

### 3.1. Overview

GazeHRNet predicts where each person in an image is looking. The input consists of an image *I* and a set of head bounding boxes $B={\left\{b_{i}\right\}}_{i=1}^{M}$, where *M* denotes the number of target persons. The model outputs a gaze heatmap for each person. For video settings, it also predicts whether the gaze target is within the frame.

**Figure 1:**
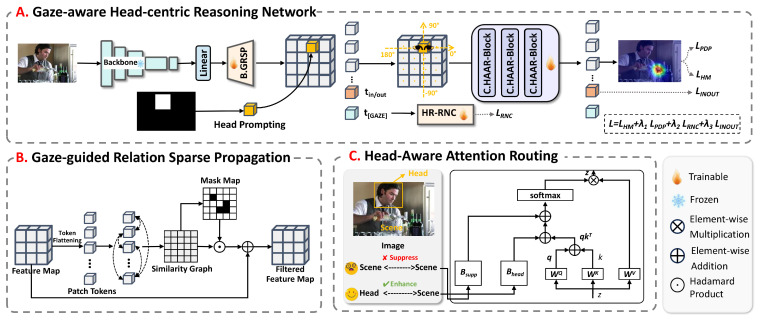
Overall architecture of the proposed GazeHRNet framework. (**A**) The full pipeline: a frozen DINOv2 backbone extracts patch features, which are processed by GRSP, conditioned with Head Prompting and HCPE, and processed by stacked HAAR blocks to produce gaze heatmap, patch distribution, and in/out predictions. The [GAZE] token receives HR-RNC constraints during training. (**B**) Gaze-guided Relation Soft Propagation: content-dependent similarity gating suppresses task-irrelevant token connections before the Transformer. (**C**) Head-Aware Attention Routing: head-guided bias $B^{\mathrm{head}}$ enhances head-scene attention flow, while suppression bias $B^{\mathrm{supp}}$ penalizes nearby background interactions.

The overall architecture is shown in Figure 1. A frozen DINOv2 backbone extracts patch-level features, which are projected to a unified dimension. GRSP filters out task-irrelevant redundancy from the image-level features before person-specific processing. Each person instance then receives head-centric spatial conditioning through HCPE and Head Prompting. The conditioned features, together with prepended [GAZE] and [INOUT] tokens, enter the HAAR Transformer for contextual modeling. The output feeds into three prediction branches: a heatmap head, a Patch Distribution Predictor (PDP), and an in/out classifier. During training, the [GAZE] token additionally receives HR-RNC constraints. A multi-task loss jointly optimizes all branches.

### 3.2. Gaze-Guided Relation Soft Propagation (GRSP)

DINOv2 features encode broad visual semantics, much of which is not useful for gaze prediction. Background textures and unrelated object features are redundant. Feeding them directly into the Transformer increases computation and may hurt accuracy. GRSP suppresses this redundancy before the Transformer stage (see Figure 1B).

The input image *I* is resized to 448×448 and fed into the frozen DINOv2 ViT-B/14, yielding patch-level features with $H_{f}=W_{f}=32$ and $C_{b}=768$ channels. A 1×1 convolution projects these to dimension D=256, producing $F\in R^{D\times H_{f}\times W_{f}}$. GRSP strengthens connections between semantically related tokens and weakens connections between unrelated ones. *F* is flattened into a token sequence $X=\left\{x_{1},\ldots ,x_{N}\right\}\in R^{N\times D}$, where $N=H_{f}\times W_{f}$.

The cosine similarity for each token pair is:

(1) $$S_{ij}=\frac{x_{i}^{⊤}x_{j}}{{∥x_{i}∥}_{2}\cdot {∥x_{j}∥}_{2}}.$$

A learnable threshold τ (initialized to 0.1) controls the gating:

(2) $$G_{ij}=\sigma \left((S_{ij}-\tau )\cdot \alpha \right),$$

where σ(·) is the sigmoid function and α=10 is a temperature coefficient. When $S_{ij}$ exceeds τ, $G_{ij}$ approaches 1; below τ, $G_{ij}$ approaches 0. The gated attention weights are normalized as:

(3) $$A_{ij}=\frac{G_{ij}\cdot \exp\left(S_{ij}/T\right)}{\sum_{k=1}^{N}G_{ik}\cdot \exp\left(S_{ik}/T\right)+\epsilon },$$

where *T* is a temperature scaling factor for the similarity scores, and ϵ is a small constant for numerical stability. This formulation ensures that the propagation weights $A_{ij}$ are strictly non-negative and properly normalized, while the learnable threshold τ effectively controls the gating behavior. The aggregated result passes through LayerNorm and a linear projection with GELU. A learnable scalar β (initialized to 0.1) controls the fusion strength:

(4) $$X^{\prime }=X+\sigma \left(\beta \right)\cdot \mathrm{Proj}\left(\mathrm{LN}(A\cdot X)\right).$$

At training start, σ(β)≈0.52, so GRSP modifies features gently to avoid disrupting pretrained representations. β adapts automatically during training. The gated sequence $X^{\prime }$ is reshaped back to $F_{g}\in R^{D\times H_{f}\times W_{f}}$. GRSP operates on image-level features before per-person conditioning, so its cost does not scale with the number of persons.

### 3.3. Head-Centric Spatial Conditioning

Gaze prediction requires the model to know both who is looking and how every spatial position relates to that person’s head. After GRSP produces the gated features $F_{g}$, each person instance is conditioned through two complementary mechanisms.

#### 3.3.1. Head Prompting

Head Prompting tells the model “who is looking” by marking the head region. For person *i* with bounding box $b_{i}=\left(x_{i}^{1},y_{i}^{1},x_{i}^{2},y_{i}^{2}\right)$ in normalized coordinates, a binary mask $H_{i}\in {\left\{0,1\right\}}^{H_{f}\times W_{f}}$ is generated at the feature map scale. A learnable vector $e_{h}\in R^{D}$ converts the mask into a prompting signal:

(5) $$F_{h}^{i}=F_{g}^{i}+H_{i}⊗e_{h},$$

where ⊗ denotes element-wise multiplication broadcast along the channel dimension.

#### 3.3.2. Head-Centric Polar Encoding (HCPE)

Head Prompting is a binary signal that marks which patches belong to the head but does not describe how other positions relate to the head spatially. The same object on the left or right side of a person may have very different gaze likelihood. Near and far regions also carry different gaze meanings. Standard absolute positional encodings cannot express this head-centric geometry.

**Figure 2:**
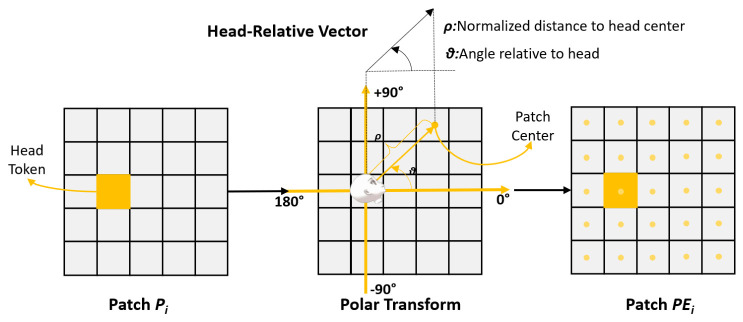
Illustration of Head-Centric Polar Encoding. For each patch $P_{i}$, HCPE computes the angle ϑ and normalized distance ρ relative to the head center, and encodes them into a per-patch positional embedding $PE_{i}$ via multi-frequency sinusoidal functions.

As illustrated in Figure 2, HCPE uses polar coordinates centered at the head to describe each patch’s relative position. For person *i*, the head center is $c_{i}=\left(x_{i}^{c},y_{i}^{c}\right)$, where $x_{i}^{c}=\left(x_{i}^{1}+x_{i}^{2}\right)/2$ and $y_{i}^{c}=\left(y_{i}^{1}+y_{i}^{2}\right)/2$. For any patch center $\left(u,v\right)\in {\left[0,1\right]}^{2}$ represented in normalized image coordinates, the direction angle $\vartheta_{i}$ and normalized distance $\rho_{i}$ are:

(6) $$\vartheta_{i}\left(u,v\right)=\mathrm{atan}2\left(v-y_{i}^{c},\;u-x_{i}^{c}\right),$$

(7) $$\rho_{i}\left(u,v\right)=\frac{\sqrt{{\left(u-x_{i}^{c}\right)}^{2}+{\left(v-y_{i}^{c}\right)}^{2}}}{\sqrt{2}}.$$

atan2(·) has output range [−π,π]. The denominator $\sqrt{2}$ is the maximum diagonal distance in normalized coordinates, ensuring $\rho_{i}\in \left[0,1\right]$.

HCPE encodes direction and distance using multi-frequency sinusoidal functions. Let K=D/4 be the number of frequency pairs with $\omega_{k}=\exp\left(-k\cdot \ln\left(10000\right)/K\right)$ for k=0,…,K−1. The encodings are:

(8) $$E_{\vartheta }=\left[\sin\left(\omega_{0}\vartheta_{i}\right),\cos\left(\omega_{0}\vartheta_{i}\right),\ldots ,\sin\left(\omega_{K-1}\vartheta_{i}\right),\cos\left(\omega_{K-1}\vartheta_{i}\right)\right]\in R^{2K},$$

(9) $$E_{\rho }=\left[\sin\left(\omega_{0}\rho_{i}\right),\cos\left(\omega_{0}\rho_{i}\right),\ldots ,\sin\left(\omega_{K-1}\rho_{i}\right),\cos\left(\omega_{K-1}\rho_{i}\right)\right]\in R^{2K}.$$

Concatenation yields $E_{i}^{\mathrm{HCPE}}=\mathrm{Concat}\left(E_{\vartheta },E_{\rho }\right)\in R^{D}$, which is added to the instance features:

(10) $$F_{p}^{i}=F_{h}^{i}+E_{i}^{\mathrm{HCPE}}.$$

Head Prompting marks local head identity. HCPE encodes global direction-distance priors. Low-frequency components capture coarse spatial variations, whereas high-frequency components encode finer differences in both direction and distance. Together, they give instance features head-centric spatial awareness before the Transformer.

### 3.4. Head-Aware Attention Routing (HAAR)

After GRSP gating and head-centric conditioning, each person instance has features $F_{p}^{i}\in R^{D\times H_{f}\times W_{f}}$. Standard self-attention does not explicitly differentiate between head, scene, and background regions. HAAR modifies self-attention with head-guided bias and background suppression to match the spatial semantics of gaze prediction (see Figure 1C).

#### 3.4.1. Sequence Construction

$F_{p}^{i}$ is flattened into $X\in R^{N\times D}$. Two learnable special tokens are prepended for VideoAttentionTarget (VAT) dataset: the [GAZE] token $t_{g}$ aggregates scene-wide gaze semantics, and the [INOUT] token $t_{io}$ handles in/out-of-frame prediction:

(11) $$Z_{\mathrm{VAT}}^{\left(0\right)}=\left[t_{io};\;t_{g};\;x_{1},\ldots ,x_{N}\right]\in R^{(N+2)\times D},$$

For GazeFollow dataset, only the [GAZE] token is prepended:

(12) $$Z_{\mathrm{GF}}^{\left(0\right)}=\left[t_{g};\;x_{1},\ldots ,x_{N}\right]\in R^{(N+1)\times D}.$$

#### 3.4.2. Head-Guided Bias

The head mask $H_{i}$ is flattened into $h\in {\left\{0,1\right\}}^{N}$. For patch positions *p* and *q*:

(13) $$B_{pq}^{\mathrm{head}}=\sigma \left(\gamma \right)\cdot \left(h_{p}+h_{q}\right),$$

where γ is learnable (initialized to 0.5). If either token belongs to the head, the pair gets a positive bias; if both do, the bias doubles.

This selectively strengthens interactions involving the head (i.e., head-to-scene, scene-to-head, and head-to-head). Bias values for special tokens are zero.

#### 3.4.3. Background Suppression

Nearby background-to-background interactions contribute noise. HAAR suppresses them with a negative bias:

(14) $$B_{pq}^{\mathrm{supp}}=-\sigma \left(\mu \right)\cdot \left(1-h_{p}\right)\left(1-h_{q}\right)\cdot 1\left[d_{\mathrm{sp}}\left(p,q\right)<R\right],$$

where μ is learnable (initialized to −1.0), R=2 is the spatial radius, and $d_{\mathrm{sp}}$ is grid Euclidean distance.

The bias activates only when both tokens are outside the head and spatially close. With μ initialized to −1.0, σ(μ)≈0.27, so the suppression starts mild and adapts during training.

##### Attention Computation

Both biases are injected into multi-head self-attention with $n_{\mathrm{heads}}=8$ and per-head dimension $d_{h}=D/n_{\mathrm{heads}}$:

(15) $$A^{\left(l\right)}=\mathrm{softmax}\;\left(\frac{Q^{\left(l\right)}{K^{\left(l\right)}}^{⊤}}{\sqrt{d_{h}}}+B^{\mathrm{head}}+B^{\mathrm{supp}}\right).$$

Each layer uses standard residual connections, a feed-forward network (expansion ratio 4, GELU), layer normalization, and DropPath (rate 0.1). HAAR stacks L=3 layers. After *L* layers, the [GAZE] token output is denoted $y_{g}=t_{g}^{\left(L\right)}\in R^{D}$.

### 3.5. Head-Relative Rank-N-Contrast Learning (HR-RNC)

**Figure 3:**
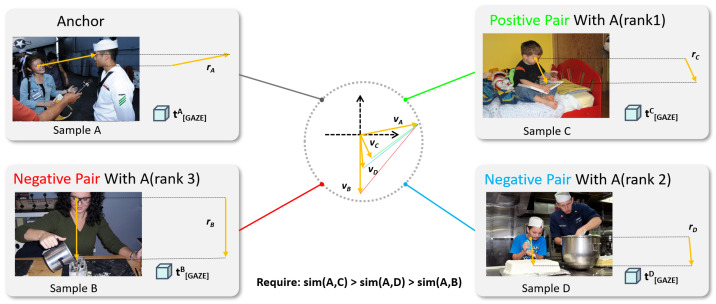
Illustration of Head-Relative Rank-N-Contrast Learning. Each sample’s head-relative gaze vector *r* defines its position in the label space. For anchor A, samples are ranked by label distance: C (rank 1) has the most similar gaze vector, followed by D (rank 2) and B (rank 3). The RnC loss requires feature similarity sim(A,C)>sim(A,D)>sim(A,B) to preserve this ordering.

Heatmap and PDP supervision constrain prediction accuracy in the output space but do not guarantee good latent space structure. Samples with similar gaze behavior may not be close in feature space. HR-RNC imposes structural constraints on the [GAZE] token output $y_{g}$ to align feature similarity with gaze behavior similarity. Figure 3 illustrates this process.

Absolute gaze coordinates $\left(g_{x},g_{y}\right)$ are a natural contrastive label choice but suffer from position bias: two persons gazing at the same target from different positions share the same gaze point yet have entirely different gaze behaviors. HR-RNC uses head-relative gaze vectors instead:

(16) $$r_{i}=g_{i}-c_{i},$$

where $g_{i}$ is the gaze point and $c_{i}$ is the head center. $r_{i}$ encodes direction and distance without the head’s absolute position. The label distance is $d_{ij}={∥r_{i}-r_{j}∥}_{2}$.

Gaze similarity is continuous, so binary positive/negative splits used in standard InfoNCE do not fit. HR-RNC adopts the Rank-N-Contrast (RnC) loss [22], which preserves the ordering of label distances without thresholds. $y_{g}^{i}$ passes through a two-layer projection head ($D\to D/2\to d_{\mathrm{proj}}$ with ReLU, $d_{\mathrm{proj}}=64$) and L2 normalization:

(17) $$z_{i}=\frac{\mathrm{Proj}(y_{g}^{i})}{∥\mathrm{Proj}\left(y_{g}^{i}\right){∥}_{2}}.$$

For each anchor *i*, all other valid samples are sorted by $d_{ij}$ in ascending order with index sequence $\pi_{i}$:

(18) $$L_{\mathrm{rnc}}=\frac{1}{B(B-2)}\sum_{i=1}^{B}\sum_{k=1}^{B-2}-\log\frac{\exp(z_{i}^{⊤}z_{\pi_{i}\left(k\right)}/T)}{\sum_{m=k}^{B-1}\exp\left(z_{i}^{⊤}z_{\pi_{i}\left(m\right)}/T\right)},$$

where *B* is the number of valid in-frame samples and T=2.0. Out-of-frame samples are excluded. When fewer than three valid samples exist, the loss is zero. The projection head is discarded at inference, adding no cost.

### 3.6. Prediction and Training

After *L* layers of HAAR, the output sequence $Z^{\left(L\right)}$ feeds into three prediction branches, each paired with its supervision signal.

#### 3.6.1. Heatmap Prediction with Anisotropic Supervision

The patch tokens are reshaped into $Y_{p}\in R^{D\times H_{f}\times W_{f}}$, upsampled via stride-2 transposed convolution, reduced to one channel by a 1×1 convolution, passed through sigmoid, and resized to produce the predicted heatmap $\hat{G}\in {\left[0,1\right]}^{64\times 64}$. The peak location is the predicted gaze target.

Existing methods use isotropic Gaussians ($\sigma_{x}=\sigma_{y}$) as heatmap labels, assuming equal uncertainty in both directions. Gaze targets typically have greater horizontal uncertainty due to the wider horizontal field of view. We use anisotropic Gaussians instead:

(19) $$G^{*}\left(u,v\right)=\exp\;\left(-\frac{{\left(u-g_{x}\right)}^{2}}{2\sigma_{x}^{2}}-\frac{{\left(v-g_{y}\right)}^{2}}{2\sigma_{y}^{2}}\right),$$

with $\sigma_{x}=3$ and $\sigma_{y}=2$. The loss is pixel-wise binary cross-entropy computed for in-frame samples: $L_{\mathrm{hm}}=\mathrm{BCE}\left(\hat{G},\;G^{*}\right)$.

#### 3.6.2. Patch Distribution Prediction

$Y_{p}$ is compressed to a P×P grid (default P=8) via adaptive average pooling. Each patch feature passes through a two-layer MLP (D→D/4→1 with GELU) to produce logit $l_{m}$ for $m=1,\ldots ,P^{2}$. A learnable outside logit $l_{\mathrm{out}}$ represents the out-of-frame case:

(20) $$\hat{p}=\mathrm{softmax}\left(\left[l_{1},\ldots ,l_{P^{2}},l_{\mathrm{out}}\right]\right)\in \Delta^{P^{2}+1}.$$

The ground-truth distribution is generated from the gaze point with a Gaussian kernel ($\sigma_{\mathrm{pdp}}=1.5$). For out-of-frame samples, all mass goes to the outside dimension. The loss is KL divergence: $L_{\mathrm{pdp}}=D_{\mathrm{KL}}\left(p\;∥\;\hat{p}\right)$.

#### 3.6.3. In/Out Prediction

On datasets with in/out annotations, the [INOUT] token output $t_{io}^{\left(L\right)}$ passes through a two-layer MLP (D→128→1, ReLU + Dropout) with sigmoid to produce $\hat{o}\in \left[0,1\right]$. The loss is binary cross-entropy: $L_{\mathrm{inout}}=\mathrm{BCE}\left(\hat{o},\;o\right)$.

#### 3.6.4. Overall Objective

(21) $$L=L_{\mathrm{hm}}+\lambda_{\mathrm{pdp}}\;L_{\mathrm{pdp}}+\lambda_{\mathrm{rnc}}\;L_{\mathrm{rnc}}+\lambda_{\mathrm{inout}}\;L_{\mathrm{inout}}.$$

Default weights are $\lambda_{\mathrm{pdp}}=0.5$, $\lambda_{\mathrm{rnc}}=0.3$, and $\lambda_{\mathrm{inout}}=1.0$. The $L_{\mathrm{inout}}$ term is omitted when the dataset has no in/out annotations. The DINOv2 backbone stays frozen. Only the GRSP, Head Prompting vector, HAAR Transformer, prediction heads, and HR-RNC projection head are optimized.

## 4. Experiments

### 4.1. Datasets and Evaluation Metrics

#### 4.1.1. Datasets

Experiments are conducted on two standard gaze target detection benchmarks: GazeFollow and VideoAttentionTarget.

GazeFollow [10] is a large-scale static-image dataset for gaze target localization. It contains 130,339 people in 122,143 images for training and 4782 people in 4782 images for testing. Each test sample is annotated by ten annotators to account for annotation ambiguity. The dataset covers diverse indoor and outdoor scenes with varying head poses and gaze distances.

VideoAttentionTarget (VAT) [11] extends gaze target detection to the video domain. It contains 1331 video sequences with 164,541 annotated frames for training and 468 sequences with 109,574 frames for testing. In addition to gaze target locations, VAT provides binary in-frame/out-of-frame labels indicating whether the gaze target is visible in the current frame. This makes VAT a more challenging benchmark that requires both spatial localization and visibility reasoning.

#### 4.1.2. Evaluation Metrics and Protocol

The following metrics are adopted following standard protocols.

AUC. The area under the ROC curve evaluates the model’s ability to rank spatial locations according to the ground-truth gaze distribution. Higher values indicate better overall localization quality.

L2 Distance. The Euclidean distance between the predicted gaze point and the ground-truth target evaluates localization accuracy. On GazeFollow, both Avg L2 (distance to the average annotation) and Min L2 (minimum distance to any annotator) are reported. Lower values indicate more accurate predictions.

AP. On VAT, average precision evaluates the performance of the binary in-frame/out-of-frame classification task. Higher values indicate better visibility prediction.

Evaluation Protocol. During testing, we strictly follow the standard protocol of utilizing ground-truth head bounding boxes to isolate gaze prediction performance from head detection errors. For images containing multiple annotated subjects, predictions are generated sequentially at the instance level. On the GazeFollow test set, where each image has 10 independent annotations, we evaluate against the aggregated ground-truth heatmap for AUC, compute Avg L2 against the arithmetic mean of the 10 coordinates, and report Min L2 as the distance to the closest annotator. Furthermore, VideoAttentionTarget is evaluated strictly as a frame-level spatial task, ensuring that no temporal consistency or motion tracking priors are utilized.

### 4.2. Implementation Details

As the official GazeFollow and VideoAttentionTarget datasets do not provide pre-defined validation splits, we randomly partitioned 10% of the training data as a separate validation set for model selection and hyperparameter tuning. The official test sets were exclusively reserved for final evaluation. Unless otherwise specified, all experiments are conducted with a fixed random seed of 0 to ensure fully deterministic initialization. The framework is implemented in PyTorch 2.5.1 and trained on a single NVIDIA RTX 5090 GPU. DINOv2 ViT-B/14 [36] serves as the frozen backbone. All input images are resized to 448×448 pixels, yielding a 32×32 feature map. The projection dimension is D=256.

The model is optimized with AdamW (weight decay $1\times 10^{-4}$). The initial learning rate is $2.5\times 10^{-4}$ with a cosine annealing schedule and 5-epoch linear warmup. The batch size is 64 on GazeFollow and 32 on VAT. Training runs for 40 epochs on GazeFollow and 30 epochs on VAT. The loss weights are $\lambda_{\mathrm{pdp}}=0.5$, $\lambda_{\mathrm{rnc}}=0.3$, and $\lambda_{\mathrm{inout}}=1.0$. The $L_{\mathrm{inout}}$ term is used only on VAT.

The GRSP module uses one layer with temperature α=10 and initial threshold τ=0.1. The HAAR Transformer has L=3 layers, $n_{\mathrm{heads}}=8$ attention heads, and a feed-forward expansion ratio of 4. DropPath rate is 0.1. The HR-RNC projection dimension is $d_{\mathrm{proj}}=64$ with temperature T=2.0. The PDP uses an 8×8 spatial grid. Anisotropic heatmap supervision uses $\sigma_{x}=3$ and $\sigma_{y}=2$ on a 64×64 output resolution.

Data augmentation includes random cropping with scale [0.8,1.0], random horizontal flipping with probability 0.5, and head bounding box jittering with a maximum offset of 5% of the box size. For VAT, frames are sampled at the original annotation rate without temporal subsampling.

### 4.3. Comparison with State-of-the-Art Methods

To ensure transparency regarding potential environment-dependent variance, we explicitly acknowledge that the quantitative results of the competing methods reported in this study are directly drawn from their original publications rather than re-run under identical conditions. Crucially, all compared methods adhere to the standardized experimental protocols, utilizing the exact same training and testing data splits provided by the official GazeFollow and VideoAttentionTarget (VAT) datasets. Furthermore, all evaluations—including the calculation of AUC and Average L2 distance—are conducted using the standard official evaluation scripts. For recent baselines where additional qualitative results were required, we utilized their official open-source codebases and pre-trained weights under consistent evaluation environments.

Table 1 compares GazeHRNet with existing methods on GazeFollow and VideoAttentionTarget. The compared methods span three categories: image-only approaches that use RGB input alone, multi-modal approaches that require auxiliary inputs such as depth maps, pose estimates, or object detections, and recent large-model-based approaches.

On GazeFollow, GazeHRNet achieves an AUC of 0.952, an Avg L2 of 0.102, and a Min L2 of 0.055. All three metrics demonstrate highly competitive performance among the compared computational models. Compared with Sharingan, which previously held the best AUC (0.944) and Avg L2 (0.113) among image-only methods, GazeHRNet reduces the Avg L2 error by 9.7%. Several multi-modal methods such as MMGaze and PatchGaze leverage depth and/or pose information but still fall behind GazeHRNet on all metrics. This indicates that head-centric spatial encoding and gaze-aware feature interaction can provide an effective alternative to explicit geometric inputs.

On VideoAttentionTarget, GazeHRNet achieves the best AUC (0.929), L2 distance (0.103), and in/out AP (0.909). The L2 distance improves upon the previous best result (0.104 by DepthGaze) while requiring no depth or pose inputs. The in/out AP of 0.909 also surpasses PatchGaze (0.908), indicating that the PDP module with its outside logit provides effective visibility reasoning. GazeVLM achieves a competitive AUC of 0.926 but falls behind on L2 and AP, suggesting that vision-language model features alone do not capture the fine-grained spatial structure that head-centric encoding provides.

Two observations are worth noting. First, GazeHRNet uses only RGB images as input yet consistently outperforms methods that rely on depth, pose, or object detection. These auxiliary inputs introduce additional preprocessing cost and potential error propagation. The head-centric design in GazeHRNet encodes geometric priors directly in the feature space without external modules. Second, the frozen DINOv2 backbone keeps the learnable parameter count low. The trainable components (GRSP, HAAR, prediction heads, and conditioning parameters) amount to approximately 3.0 M parameters, which is substantially fewer than most competing methods.

### 4.4. Cross-Dataset Generalization

To evaluate generalization ability, we train GazeHRNet on GazeFollow and directly test it on three unseen datasets without fine-tuning: VideoAttentionTarget (VAT), GOO-Real [50], and ChildPlay. Table 2 reports the results.

GazeHRNet achieves the best AUC and L2 on all three datasets. On GOO-Real, which contains grocery-store scenes that differ substantially from the training distribution, GazeHRNet reaches an AUC of 0.892 and an L2 of 0.173. This improves over PatchGaze by 2.3% in AUC and 14.4% in L2, despite PatchGaze using depth as an additional input. On ChildPlay, which focuses on children whose head proportions and gaze patterns differ from adults, GazeHRNet achieves an AUC of 0.939 and an L2 of 0.109. On VAT, the cross-dataset AUC of 0.928 closely matches the in-domain result (0.929 in Table 1), indicating minimal domain gap for temporal scenes.

These results suggest that head-centric polar encoding contributes to the generalization across domains. Because HCPE represents spatial structure relative to the head rather than in absolute coordinates, the learned features transfer to new scene layouts and subject populations without retraining.

To further validate the reliability of our proposed method, we retrained the model using 10 different random seeds (ranging from 1 to 10) to assess its statistical stability. The resulting average performance on GazeFollow is AUC 0.951±0.001, Avg L2 0.103±0.001, and Min L2 0.056±0.001. Similarly, on VideoAttentionTarget, the model yields an average AUC of 0.927±0.001, L2 of 0.104±0.001, and AP of 0.908±0.002. These negligible standard deviations demonstrate the robustness and reproducibility of our results, indicating that our performance gains are highly stable across different random initializations. Since the original instance-level predictions of the baselines are unavailable, a direct paired significance test cannot be conducted. However, the performance margin over the strongest baselines (e.g., Sharingan’s 0.944 AUC on GazeFollow) is substantially larger than our model’s standard deviation. This statistical confidence indicates that the performance gain, though numerically small, is highly stable and well outside the margin of random error. Furthermore, this exceptionally low standard deviation reflects the true training stability of our framework rather than an artifact of the experimental setup. This stability stems primarily from relying on a frozen, highly robust DINOv2 backbone and optimizing only a very lightweight (3 M parameters) gaze-specific decoder, which significantly simplifies the optimization landscape. We confirm that the random seeds strictly controlled all stochastic processes during training, including data shuffling, random cropping, and weight initialization for the trainable decoder.

### 4.5. Ablation Study

To verify the contribution of each proposed module, we conduct a block-wise ablation study on GazeFollow. Table 3 organizes the experiments into three groups, each addressing one core question: whether head-centric spatial encoding provides useful geometric priors (No. 1–3), whether gaze-aware feature interaction modules are independently effective and complementary (No. 4–9), and whether coarse-to-fine supervision and head-relative contrastive learning improve localization and representation quality (No. 10–14). Within each group, individual and combined configurations are evaluated through controlled module combinations. The baseline (No. 0) retains only the frozen DINOv2 backbone, a linear projection, binary Head Prompting, standard 2D sinusoidal positional encoding, a three-layer vanilla self-attention Transformer, a convolutional decoder, and isotropic Gaussian heatmap supervision.

Direction encoding $E_{\vartheta }$ alone (No. 1) raises AUC from 0.876 to 0.884, and distance encoding $E_{\rho }$ alone (No. 2) raises AUC to 0.881. Both improve the baseline independently, confirming that angular orientation and radial distance provide distinct head-relative geometric cues. Direction encoding yields a slightly larger standalone gain (+0.008 vs. +0.005 in AUC), consistent with gaze localization being primarily constrained by viewing orientation. Combining both (No. 3) raises AUC to 0.895, outperforming either component alone and confirming that direction and distance are complementary.

The feature interaction group (No. 4–9) contributes the largest group gain (+0.034 AUC over No. 3). GRSP alone (No. 4) raises AUC to 0.904 by filtering gaze-irrelevant token relations before the Transformer. Among the two routing biases, head-guided bias $B^{\mathrm{head}}$ (No. 5, AUC 0.913) provides a larger improvement than background suppression $B^{\mathrm{supp}}$ (No. 6, AUC 0.908), indicating that strengthening head-scene communication matters more than suppressing background interactions. Combining GRSP with either bias (No. 7 and No. 8) consistently outperforms the bias-only counterparts (No. 5 and No. 6), with GRSP contributing a stable +0.009–0.010 AUC regardless of HAAR configuration. This stability suggests that GRSP and HAAR operate at complementary stages. The full interaction module (No. 9, AUC 0.929) outperforms both No. 7 (0.923) and No. 8 (0.918), confirming that head-guided enhancement and background suppression are complementary rather than redundant.

PDP alone (No. 10) raises AUC to 0.933 by providing coarse 8×8 patch-level spatial guidance. AHS alone (No. 11, AUC 0.935) produces a larger reduction in localization distance (Avg L2 from 0.154 to 0.137, vs. 0.145 for PDP), confirming that elliptical labels better capture the directional variance of gaze annotations. Combining both (No. 12) raises AUC to 0.939 and reduces Avg L2 to 0.126, validating the coarse-to-fine supervision design. Adding the Rank-N-Contrast loss with absolute gaze coordinates (No. 13) raises AUC to 0.944 by imposing ordinal structure on the [GAZE] token latent space. Switching the label space from absolute coordinates to head-relative gaze vectors (No. 14) yields the largest single-step gain in the entire table (AUC from 0.944 to 0.952, Min L2 from 0.066 to 0.055), because head-relative vectors remove the confound between absolute position and gaze behavior. No. 13 and No. 14 differ in exactly one factor—the label space $g_{i}$ vs. $r_{i}=g_{i}-c_{i}$—making this a controlled comparison that supports the head-relative formulation. The total gain from baseline to full model is +0.076 AUC, with HCPE contributing +0.019, feature interaction +0.034, and supervision and representation +0.023.

To comprehensively evaluate module interactions and verify that cumulative ablations do not underestimate them, we conducted a complementary removal-from-full ablation analysis (Table 4). Starting from the full GazeHRNet, we individually removed each core component. The results show that removing HAAR causes the most severe performance drop (AUC decreases from 0.952 to 0.932), highlighting its critical role in feature interaction. Removing HCPE or HR-RNC also leads to a clear performance drop, indicating that head-centric spatial encoding and ordinal representation constraints are essential. Furthermore, removing grouped modules (e.g., both HCPE and HAAR) results in compounding errors, supporting the interpretation that these components provide complementary benefits to the final architecture.

### 4.6. Hyperparameter Sensitivity

**Figure 4:**
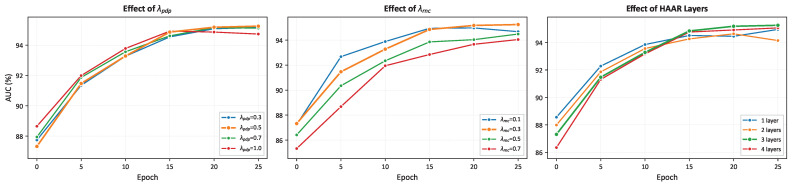
Hyperparameter sensitivity analysis on GazeFollow. **Left:** effect of PDP loss weight $\lambda_{\mathrm{pdp}}$; $\lambda_{\mathrm{pdp}}=0.5$ achieves the best final AUC, while larger weights accelerate early convergence but cause late-stage degradation. **Middle:** effect of HR-RNC loss weight $\lambda_{\mathrm{rnc}}$; the model is more sensitive to this parameter, with $\lambda_{\mathrm{rnc}}=0.3$ best balancing representation structure and localization accuracy. **Right:** effect of the number of HAAR Transformer layers; three layers yield the best trade-off between capacity and optimization efficiency.

We examine the sensitivity of GazeHRNet to three key hyperparameters on GazeFollow: the PDP loss weight $\lambda_{\mathrm{pdp}}$, the HR-RNC loss weight $\lambda_{\mathrm{rnc}}$, and the number of HAAR Transformer layers. All other settings are kept identical. Figure 4 shows AUC curves over training epochs.

For $\lambda_{\mathrm{pdp}}$ (Figure 4, left), $\lambda_{\mathrm{pdp}}=0.5$ achieves the best final AUC. Larger weights accelerate early convergence but hurt final performance because the coarse distribution constraint begins to dominate the overall optimization objective. The final AUC varies by only about 0.5% across all tested values, indicating robustness to this parameter. For $\lambda_{\mathrm{rnc}}$ (Figure 4, middle), the model is more sensitive: $\lambda_{\mathrm{rnc}}=0.3$ gives the best result, while too-small weights lead to late-stage degradation and too-large weights significantly hurt performance throughout training. This suggests that HR-RNC should serve as a moderate auxiliary signal rather than a dominant training objective. For the number of HAAR layers (Figure 4, right), three layers achieve the best final AUC. One layer converges fastest initially but saturates early due to limited representational capacity, while four layers introduce redundant parameters without further improvement.

To provide a more comprehensive analysis of key hyperparameters, we conduct additional sensitivity evaluations on the GazeFollow dataset. First, we examine the anisotropic heatmap variances ($\sigma_{x}$, $\sigma_{y}$). As shown in Table 5, we evaluate various combinations of $\sigma_{x}$ and $\sigma_{y}$ to validate our empirical choice of $\sigma_{x}=3,\sigma_{y}=2$. Second, we evaluate the sensitivity of the GRSP parameters, specifically the initial threshold τ and the temperature α, which control the feature filtering process (Table 6). Finally, we analyze the impact of the temperature *T* in the HR-RNC loss on the learned [GAZE] token representation (Table 7). All experiments use the primary metrics of AUC, Avg L2, and Min L2.

The results reveal key insights into the model design. For the anisotropic heatmap (Table 5), the optimal configuration ($\sigma_{x}=3,\sigma_{y}=2$) shows that the model favors $\sigma_{x}>\sigma_{y}$. This aligns with the physiological prior that human horizontal gaze shifts are more frequent and exhibit larger variance than vertical shifts, requiring greater spatial tolerance along the X-axis. Furthermore, we investigated whether making these parameters learnable during optimization could improve performance. By implementing a differentiable heatmap generation process and introducing global learnable parameters for $\sigma_{x}$ and $\sigma_{y}$, we observed a slight performance drop (AUC = 0.923). This indicates that freely learning the variance causes the supervision target to drift during training, whereas the fixed predefined prior provides a crucial regularization effect for stable convergence. For the GRSP parameters (Table 6), τ=0.10 and α=10 achieve the best performance. A lower τ fails to effectively prune dense, noisy relations, while a higher τ overly sparsifies the graph, discarding valuable contextual cues. Similarly, α=10 provides the ideal sharpness for the gating sigmoid function, avoiding the gradient vanishing issues of a hard step function. Finally, the HR-RNC temperature T=2.0 (Table 7) provides the optimal balance for contrastive learning. Lower temperatures make the distribution excessively sharp and unstable, heavily penalizing hard negative samples, whereas higher temperatures over-smooth the logits, reducing discriminability. Regarding the batch construction for the HR-RNC loss, within a default batch size of 64 on GazeFollow, out-of-frame samples are filtered out, resulting in an average of 50 to 60 valid in-frame samples per batch. This provides sufficient positive and negative pairs for the rank-based contrastive objective. We also empirically verified that the HR-RNC module is highly robust to varying batch sizes. For instance, testing with batch sizes of 32, 64, and 128 yielded AUC scores of 0.950, 0.952, and 0.951 respectively, confirming that the representation learning is stable as long as the batch contains a reasonable number of valid samples.

### 4.7. Qualitative Results

**Figure 5:**
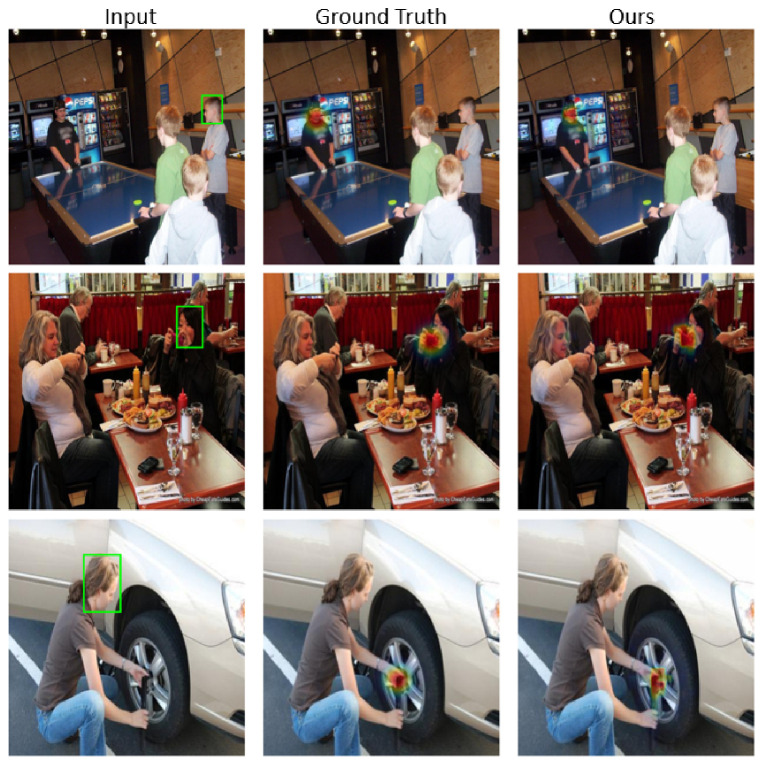
Qualitative results on GazeFollow. Each row shows one example. **Left:** input image with the target person’s head marked by a green bounding box. **Middle:** ground-truth gaze heatmap. **Right:** GazeHRNet prediction. For both ground-truth and predictions, red circles indicate the annotated/predicted gaze target locations, while the overlaid colored heatmaps visualize the probability distributions. The model accurately localizes gaze targets in multi-person, cluttered, and object-interaction scenarios.

Figure 5 shows qualitative results of GazeHRNet on GazeFollow. The predicted heatmaps closely match the ground-truth gaze distributions across diverse scenarios, including multi-person scenes with occlusion (row 1), cluttered dining environments with multiple candidate objects (row 2), and outdoor scenes where the gaze target is a manipulated object rather than a visually salient region (row 3). In all cases, GazeHRNet produces concentrated responses around the true gaze target without being distracted by irrelevant salient objects.

**Figure 6:**
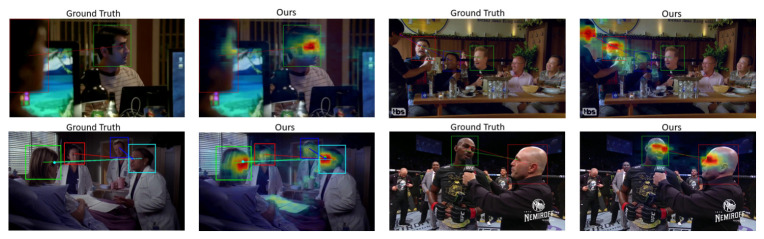
Qualitative generalization results of GazeHRNet in challenging real-world environments. In each image, colored bounding boxes denote the detected heads of different subjects, and the corresponding solid lines of the same color point from the head center to their predicted gaze target locations (highlighted by the overlaid heatmaps). The model successfully infers gaze targets in scenarios involving severe head occlusions (top-left), complex multi-person social interactions (top-right and bottom-left), and dynamic sporting events with intense mutual focus (bottom-right).

To further validate the generalization ability of GazeHRNet beyond standard benchmark settings, we conducted additional qualitative evaluations in highly challenging real-world environments. As illustrated in Figure 6, the model was tested in scenarios featuring severe head occlusions, dense multi-person social interactions, and dynamic environments (e.g., sports interviews). Despite the presence of multiple interacting subjects and cluttered backgrounds, GazeHRNet consistently produces accurate and concentrated gaze heatmaps. Notably, in cases of mutual gaze or complex social dynamics (e.g., Figure 6, bottom-left), the model effectively distinguishes the correct target person from other visually salient candidates. These results strongly demonstrate that our head-centric spatial conditioning and global relationship modeling generalize well to unconstrained, complex real-world applications.

**Figure 7:**
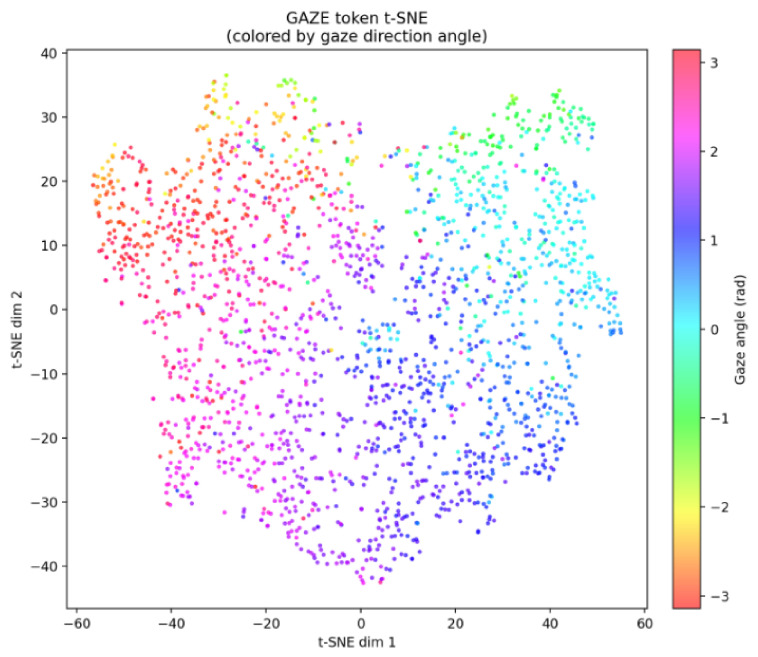
t-SNE visualization of [GAZE] token features on the GazeFollow test set, colored by head-relative gaze direction angle (in radians). The smooth color gradient from left-looking (warm) to right-looking (cool) indicates that HR-RNC organizes the feature space according to gaze direction rather than absolute position.

To verify that HR-RNC learns a structured representation of gaze behavior, we provide an illustrative t-SNE visualization of the [GAZE] token features from the GazeFollow test set, colored by head-relative gaze direction angle. As shown in Figure 7, the embedding space exhibits a smooth angular gradient: left-looking samples (warm colors) cluster on the left, while right-looking samples (cool colors) cluster on the right. This suggests that the Rank-N-Contrast objective with head-relative labels successfully induces an ordinal structure aligned with gaze direction in the feature space, rather than memorizing absolute spatial positions.

**Figure 8:**
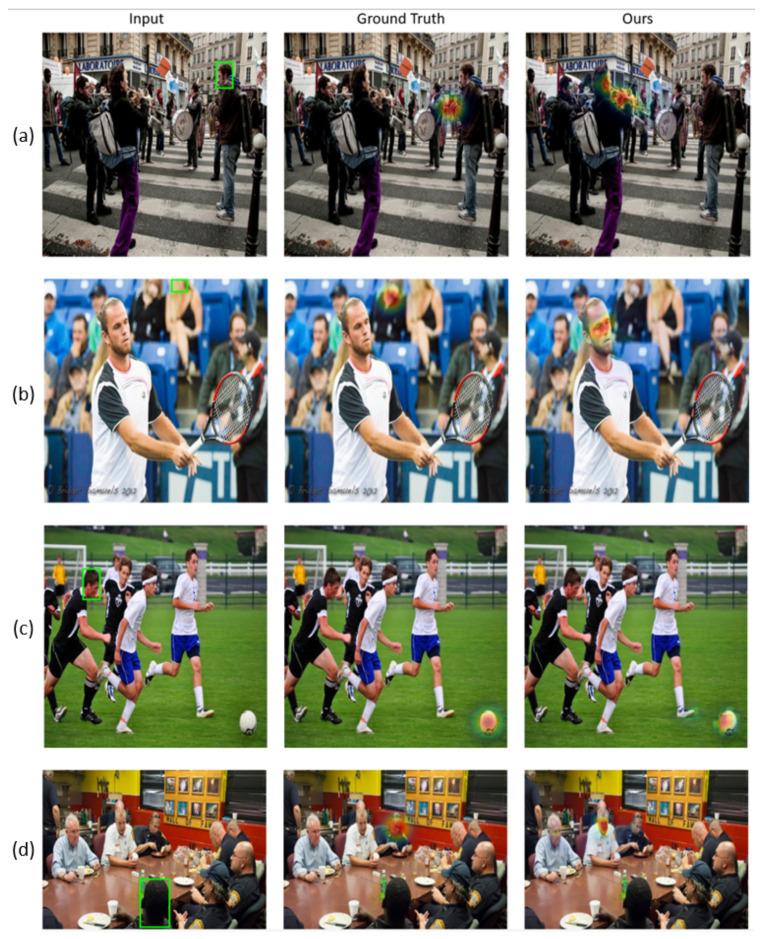
Typical failure cases of GazeHRNet categorized into four scenarios. The three columns show the input image, the ground-truth gaze heatmap, and the gaze heatmap predicted by GazeHRNet, respectively. In the input images, the green rectangles indicate the head bounding boxes of the target persons. In the ground-truth and predicted heatmaps, warmer colors, such as red and yellow, indicate higher gaze likelihood, whereas cooler colors, such as green and blue, indicate lower gaze likelihood. The colored circular regions represent gaze heatmap responses rather than additional annotations. From top to bottom: (**a**) crowded scenes causing dispersed attention; (**b**) extremely small head regions leading to feature loss; (**c**) incorrect gaze direction estimation due to complex body posture; and (**d**) severe head occlusion forcing the model to over-rely on contextual guessing.

Figure 8 shows four representative failure cases categorized by their primary challenges. In crowded scenes (Figure 8a), although the model identifies the general target area, the presence of multiple interacting subjects and complex backgrounds causes the predicted heatmap to become dispersed. For extremely small head regions (Figure 8b), the low resolution leads to a severe loss of facial features, forcing the model to rely on global context and resulting in imprecise predictions. In dynamic scenarios like sports (Figure 8c), complex body postures can mislead the model’s gaze direction estimation, sometimes creating multiple peaks. Finally, under severe head occlusion or when subjects face completely away from the camera (Figure 8d), the absence of explicit directional cues forces the network to over-rely on body posture and scene priors, which often produces unreliable artifacts.

To gain deeper insights into these limitations, we systematically categorized common failure cases on the GazeFollow test set into these four main types. For each category, we randomly sampled 100 challenging images for detailed visual inspection. This structured review confirmed that dense crowds with severe mutual occlusion and extremely small head regions remain the most persistent challenges, as they provide limited directional cues and multiple visually similar candidate objects compete for attention. Incorporating higher-resolution head features or explicit occlusion reasoning may help address such cases in the future.

### 4.8. Robustness to Head Bounding Box Perturbations

To comprehensively validate the stability of GazeHRNet against inaccurate head localization, we conducted detailed perturbation experiments on the GazeFollow test set. We introduced systematic noise (translation, scaling, and their combination) to the ground-truth head bounding boxes at varying intensity levels from 5% up to an extreme 20%. To account for the randomness of the perturbations and demonstrate statistical stability, the evaluation at each non-zero noise level was repeated across 10 different random seeds, and we report the mean and standard deviation of the performance metrics (Table 8).

The experimental results demonstrate that our framework exhibits strong robustness to spatial localization noise. Even under a severe 20% combined perturbation applied to the ground-truth boxes, the AUC metric experienced a negligible drop, and the average L2 distance showed a marginal relative increase of less than 1% (from 0.1020 to 0.1028), with extremely small variance across random seeds. This strong robustness suggests that our head-centric spatial conditioning and global relationship modeling effectively capture contextual semantics without being overly sensitive to precise head cropping.

### 4.9. Impact of Frozen Visual Backbones

To demonstrate the general applicability of our proposed head-centric framework across different feature extractors, we evaluated GazeHRNet on the GazeFollow dataset using various representative frozen visual backbones, including ResNet-50, ResNet-152, MAE ViT-B/16 [37], DINOv1 ViT-B/16 [35], and EVA-02 ViT-B/16 [51]. As shown in Table 9, our proposed modules consistently yield strong performance regardless of the chosen backbone. Notably, DINOv2 ViT-B/14 remains the optimal choice, achieving the highest AUC (0.952). We attribute this to DINOv2’s self-supervised objective, which specifically targets dense, patch-level spatial reasoning and semantic consistency. Additionally, its higher spatial resolution (32×32 grid for a 448×448 input) naturally benefits fine-grained localization compared to the 28×28 grid produced by ViT-B/16 models.

### 4.10. Computational Efficiency Analysis

A comprehensive evaluation of computational efficiency is crucial for modern gaze estimation architectures. Evaluated on a single NVIDIA RTX 5090 GPU (input resolution 448×448, batch size 1), our model achieves a highly efficient real-time inference speed of 65.5 FPS (15.27 ms per frame) with an overall computational complexity of 96.0G MACs. Notably, while the model comprises 89.5M parameters in total, only 3.0M are trainable due to the frozen DINOv2 backbone.

Table 10 presents a component-wise inference time breakdown. The backbone feature extraction naturally accounts for the majority of the latency (69.74%). In contrast, our proposed core components—including Gaze-guided Relation Soft Propagation (GRSP), Head-Centric Spatial Conditioning, and Head-Aware Attention Routing (HAAR)—are highly lightweight. While they collectively consume approximately 28% of the total inference time, this slight increase in latency is well within acceptable margins for real-time applications given the significant performance gains they deliver. This demonstrates that our head-centric decoder design strikes an excellent balance between state-of-the-art accuracy and real-time efficiency.

## 5. Conclusions

This paper presents GazeHRNet, a head-centric reasoning framework for RGB-based gaze target detection. Instead of treating gaze prediction as a generic heatmap localization problem, we revisit the task from its intrinsic spatial structure: the attended target should be understood with respect to the gazer’s head. Based on this perspective, GazeHRNet organizes scene features in a head-centered manner, enhances head-scene interaction through explicit spatial biases, and learns gaze representations that reflect relative viewing behavior rather than absolute image positions. This design enables the model to reason about direction, distance, and spatial relevance without relying on additional depth, pose, or object-level annotations.

Extensive experiments on GazeFollow and VideoAttentionTarget demonstrate that GazeHRNet achieves state-of-the-art results across all major metrics using only RGB input and a small number of trainable parameters. Cross-dataset evaluation on VAT, GOO-Real, and ChildPlay further shows that the head-centric formulation generalizes well across different scene layouts and subject distributions. Ablation studies confirm that each component contributes independently, with the feature interaction group providing the largest group gain (+0.034 AUC) and the head-relative label space yielding the largest single-step improvement. Nevertheless, challenging cases remain when the head region is extremely small or heavily occluded, where directional cues become unreliable and multiple candidate targets compete for attention.

Beyond standard benchmark evaluations, GazeHRNet exhibits strong potential for integration into real-world downstream applications. In human–robot collaboration and intelligent manufacturing, our model can act as an upstream visual perception module to infer human attention, thereby enabling systems to anticipate human intentions and provide real-time assembly assistance. For assistive systems, it can facilitate hands-free interaction for individuals with motor impairments by reliably localizing gaze targets in unconstrained environments. Regarding deployment considerations, while the DINOv2 backbone accounts for the majority of the computational footprint, our custom head-centric decoder modules are exceptionally lightweight, adding negligible latency. For practical deployment on edge devices, the frozen ViT backbone can be heavily optimized using hardware acceleration libraries (e.g., TensorRT) or model quantization (e.g., FP16/INT8) without retraining our proposed task-specific modules. This decoupled architecture inherently simplifies the deployment pipeline and ensures that GazeHRNet can effectively support intelligent, human-centered systems in the wild.

However, we acknowledge several limitations in the current framework. First, as a purely spatial model, it lacks temporal consistency modeling, which could be beneficial for tracking gaze in continuous video streams. Second, the reliance on annotated or detected head bounding boxes means that performance remains bounded by the quality of upstream head detection, particularly in datasets with inherent biases or under extreme conditions involving tiny head regions and severe occlusion. Furthermore, as gaze inference technologies advance, they raise important ethical and privacy concerns. Inferring human attention without explicit consent could lead to unintended surveillance or privacy infringements. Future deployments of gaze target detection systems must ensure transparency, obtain informed user consent, and incorporate privacy-preserving mechanisms.

Future work will explore higher-resolution head modeling, temporal consistency, and explicit occlusion-aware reasoning to further improve gaze target detection in complex real-world scenarios. 

## Figures and Tables

**Table 1:** Comparison with state-of-the-art methods on GazeFollow and VideoAttentionTarget. All baseline methods report their fully trainable total parameters. For our GazeHRNet, we report the trainable parameters (3.0 M), while the total parameter count, including the frozen DINOv2 backbone, is 89.5 M. The Input column indicates auxiliary modalities: I = image, D = depth, P = pose, O = objects, E = eyes. **Bold** marks the best result and underline marks the second best. The up arrow (↑) indicates that higher values are better, and the down arrow (↓) indicates that lower values are better.

			GazeFollow			VideoAttentionTarget		
Method	Params	Input	AUC ↑	Avg L2 ↓	Min L2 ↓	AUC ↑	L2 ↓	AP ↑
One Human	–	–	0.924	0.096	0.040	0.921	0.051	0.925
SalGaze [8]	50 M	I	0.878	0.190	0.113	–	–	–
GASE [44]	51 M	I	0.896	0.187	0.112	0.833	0.171	0.712
GazeDir [9]	55 M	I	0.906	0.145	0.081	–	–	–
DAT [11]	61 M	I	0.921	0.137	0.077	0.860	0.134	0.853
JMCGaze [45]	50 M	I	0.908	0.136	0.074	–	–	–
DualAttn [12]	68 M	I+D+E	0.922	0.124	0.067	0.905	0.108	0.896
ESCNet [23]	29 M	I+D+P	0.928	0.122	–	0.885	0.120	0.869
DepthGaze [14]	52 M	I+D+P	0.920	0.118	0.063	0.900	0.104	0.895
MMACD [46]	92 M	I+D	0.927	0.141	–	0.862	0.125	0.742
IAGaze [7]	61 M	I+D+O	0.923	0.128	0.069	0.880	0.118	0.881
MMGaze [13]	35 M	I+D+P	0.943	0.114	0.056	0.914	0.110	0.879
Gaze3D [47]	46 M	I+D	0.896	0.196	0.127	0.832	0.199	0.800
PatchGaze [20]	61 M	I+D	0.934	0.123	0.065	0.917	0.109	0.908
ChildPlay [48]	25 M	I+D	0.939	0.122	0.062	0.914	0.109	0.834
Sharingan [24]	135 M	I	0.944	0.113	0.057	–	0.107	0.891
GazeVLM [49]	–	I	0.929	0.131	0.076	0.926	0.112	0.898
**GazeHRNet (Ours)**	3.0 M	I	**0.952**	**0.102**	**0.055**	**0.929**	**0.103**	**0.909**

**Table 2:** Cross-dataset evaluation. All models are trained on GazeFollow and tested directly on VAT, GOO-Real, and ChildPlay without fine-tuning. **Bold** marks the best result and underline marks the second best. The up arrow (↑) indicates that higher values are better, and the down arrow (↓) indicates that lower values are better.

	VAT		GOO-Real		ChildPlay	
Method	AUC ↑	L2 ↓	AUC ↑	L2 ↓	AUC ↑	L2 ↓
DAT	0.906	0.119	0.670	0.334	0.912	0.121
DepthGaze	0.900	0.104	–	–	–	–
MMACD	–	–	0.840	0.238	–	–
PatchGaze	0.923	0.109	0.869	0.202	0.933	0.113
MMGaze	0.907	0.137	–	–	0.923	0.142
ChildPlay	0.911	0.123	–	–	0.932	0.115
**GazeHRNet (Ours)**	**0.928**	**0.103**	**0.892**	**0.173**	**0.939**	**0.109**

**Table 3:** Block-wise ablation study of GazeHRNet on GazeFollow. No. 1–3 verify head-centric spatial encoding. No. 4–9 verify gaze-aware feature interaction. No. 10–14 verify supervision and representation learning. Within each group, individual and complementary effects are evaluated using controlled module combinations. **Bold** marks the best result within each group and the overall best result. The up arrow (↑) indicates that higher values are better, and the down arrow (↓) indicates that lower values are better. The checkmark (✓) indicates the inclusion of a specific module.

	HCPE			HAAR			Supervision & Repr.				GazeFollow		
No.	$E_{\vartheta }$	$E_{\rho }$	GRSP	[GAZE]	$B^{\mathrm{head}}$	$B^{\mathrm{supp}}$	PDP	AHS	RnC	HR	AUC ↑	Avg L2 ↓	Min L2 ↓
0											0.876	0.245	0.187
1	✓										0.884	0.226	0.164
2		✓									0.881	0.231	0.169
**3**	✓	✓									**0.895**	**0.209**	**0.145**
4	✓	✓	✓								0.904	0.193	0.132
5	✓	✓		✓	✓						0.913	0.181	0.116
6	✓	✓		✓		✓					0.908	0.187	0.123
7	✓	✓	✓	✓	✓						0.923	0.168	0.099
8	✓	✓	✓	✓		✓					0.918	0.174	0.107
**9**	✓	✓	✓	✓	✓	✓					**0.929**	**0.154**	**0.086**
10	✓	✓	✓	✓	✓	✓	✓				0.933	0.145	0.078
11	✓	✓	✓	✓	✓	✓		✓			0.935	0.137	0.075
12	✓	✓	✓	✓	✓	✓	✓	✓			0.939	0.126	0.071
13	✓	✓	✓	✓	✓	✓	✓	✓	✓		0.944	0.114	0.066
**14**	✓	✓	✓	✓	✓	✓	✓	✓	✓	✓	**0.952**	**0.102**	**0.055**

No. 0: Baseline. No. 1–3: Verify head-centric spatial encoding. No. 4–9: Verify gaze-aware feature interaction. No. 10–14: Verify supervision and representation learning. $E_{\vartheta }$: Direction encoding. $E_{\rho }$: Distance encoding. GRSP: Gaze-guided Relation Soft Propagation. [GAZE]: Learnable gaze aggregation token. $B^{\mathrm{head}}$: Head-guided attention bias. $B^{\mathrm{supp}}$: Background suppression bias. PDP: Patch Distribution Predictor. AHS: Anisotropic Heatmap Supervision. RnC: Rank-N-Contrast loss with absolute gaze coordinates. HR: Head-relative gaze vector label space (replacing absolute coordinates in RnC).

**Table 4:** Removal-from-full ablation analysis on the GazeFollow dataset. Each row represents the full model with the specified component(s) removed. **Bold** marks the best result. The up arrow (↑) indicates that higher values are better, and the down arrow (↓) indicates that lower values are better.

Configuration	AUC ↑	Avg L2 ↓	Min L2 ↓
w/o HCPE	0.938	0.125	0.075
w/o GRSP	0.942	0.115	0.071
w/o HAAR	0.932	0.137	0.090
w/o PDP	0.948	0.113	0.059
w/o AHS	0.946	0.121	0.062
w/o HR-RNC	0.939	0.126	0.071
w/o HCPE & HAAR	0.915	0.166	0.106
w/o PDP & AHS	0.942	0.130	0.070
**Full GazeHRNet**	**0.952**	**0.102**	**0.055**

**Table 5:** Sensitivity analysis of anisotropic heatmap parameters ($\sigma_{x},\sigma_{y}$) on GazeFollow. **Bold** marks the best result.

		$\sigma_{x}$				
		1	2	3	4	5
	1	0.939	0.945	0.950	0.948	0.944
$\sigma_{y}$	2	0.946	0.949	**0.952**	0.951	0.947
	3	0.942	0.943	0.949	0.948	0.943
	4	0.935	0.938	0.944	0.942	0.939

**Table 6:** Sensitivity analysis of GRSP parameters (τ and α) on GazeFollow. **Bold** marks the best result. The up arrow (↑) indicates that higher values are better, and the down arrow (↓) indicates that lower values are better.

τ	AUC ↑	Avg L2 ↓	Min L2 ↓	α	AUC ↑	Avg L2 ↓	Min L2 ↓
0.05	0.948	0.103	0.057	5	0.950	0.102	0.057
0.10	**0.952**	**0.102**	**0.055**	10	**0.952**	**0.102**	**0.055**
0.15	0.946	0.107	0.060	15	0.951	0.103	0.056
0.20	0.948	0.104	0.056	20	0.948	0.106	0.061

**Table 7:** Sensitivity analysis of HR-RNC temperature *T* on GazeFollow. **Bold** marks the best result. The up arrow (↑) indicates that higher values are better, and the down arrow (↓) indicates that lower values are better.

*T*	AUC ↑	Avg L2 ↓	Min L2 ↓
0.5	0.940	0.122	0.069
1.0	0.945	0.113	0.062
1.5	0.949	0.103	0.057
2.0	**0.952**	**0.102**	**0.055**
2.5	0.950	0.102	0.054
3.0	0.947	0.105	0.058

**Table 8:** Robustness of GazeHRNet to ground-truth head bounding box perturbations on the GazeFollow test set. Results are averaged over 10 random seeds per noise level. The up arrow (↑) indicates that higher values are better, and the down arrow (↓) indicates that lower values are better.

Perturbation	Noise Ratio	AUC (Mean ± Std) ↑	Avg L2 (Mean ± Std) ↓
None (Original)	0.00	0.9520	0.1020
Translation	0.05	0.9520±0.0001	0.1021±0.0003
	0.10	0.9518±0.0002	0.1023±0.0005
	0.15	0.9516±0.0003	0.1026±0.0007
	0.20	0.9514±0.0005	0.1028±0.0010
Scaling	0.05	0.9520±0.0001	0.1021±0.0001
	0.10	0.9520±0.0002	0.1021±0.0002
	0.15	0.9520±0.0003	0.1022±0.0004
	0.20	0.9519±0.0004	0.1023±0.0006
Combined	0.05	0.9519±0.0001	0.1021±0.0004
	0.10	0.9518±0.0003	0.1023±0.0007
	0.15	0.9515±0.0004	0.1026±0.0010
	0.20	0.9512±0.0006	0.1028±0.0012

**Table 9:** Performance comparison of GazeHRNet using different frozen visual backbones on the GazeFollow dataset. **Bold** marks the best result. The up arrow (↑) indicates that higher values are better, and the down arrow (↓) indicates that lower values are better.

Frozen Backbone	AUC ↑	Avg L2 ↓	Min L2 ↓
ResNet-50	0.901	0.198	0.138
ResNet-152	0.919	0.178	0.117
MAE ViT-B/16	0.928	0.157	0.106
DINOv1 ViT-B/16	0.931	0.149	0.102
EVA-02 ViT-B/16	0.946	0.110	0.061
**DINOv2 ViT-B/14 (Ours)**	**0.952**	**0.102**	**0.055**

**Table 10:** Component-wise efficiency metrics of GazeHRNet. Measurements are averaged over 100 runs on a single NVIDIA RTX 5090 GPU with 448×448 input resolution and batch size 1. **Bold** marks the best result.

Component	Params (M)	MACs (G)	Memory (MB)	Time (ms)
Backbone (DINOv2)	86.5	94.0	456.0	10.65
GRSP Module	0.0	<0.1	<1.0	0.82
HCPE Module	0.0	<0.1	<1.0	0.34
HAAR Module	3.0	1.8	8.0	3.08
Prediction Heads	<0.1	<0.1	<1.0	0.38
**Overall**	**89.5**	**96.0**	**∼466.0**	**15.27**

## Data Availability

The datasets used in this study, GazeFollow and VideoAttentionTarget, are publicly available from their respective original sources. The source code of GazeHRNet is available at https://github.com/Zhikanyoumai/gazehrnet (accessed on 16 July 2026).

## Abbreviations

The following abbreviations are used in this manuscript:

HCPE	Head-Centric Polar Encoding
GRSP	Gaze-guided Relation Sparse Propagation
HAAR	Head-Aware Attention Routing
PDP	Patch Distribution Predictor
AHS	Anisotropic Heatmap Supervision
HR-RNC	Head-Relative Rank-N-Contrast Learning
ViT	Vision Transformer
